# A Complex Genomic Rearrangement Involving the *Endothelin 3* Locus Causes Dermal Hyperpigmentation in the Chicken

**DOI:** 10.1371/journal.pgen.1002412

**Published:** 2011-12-22

**Authors:** Ben Dorshorst, Anna-Maja Molin, Carl-Johan Rubin, Anna M. Johansson, Lina Strömstedt, Manh-Hung Pham, Chih-Feng Chen, Finn Hallböök, Chris Ashwell, Leif Andersson

**Affiliations:** 1 Science for Life Laboratory, Department of Medical Biochemistry and Microbiology, Uppsala University, Uppsala, Sweden; 2 Science for Life Laboratory, Department of Animal Breeding and Genetics, Swedish University of Agricultural Sciences, Uppsala, Sweden; 3 Department of Animal Science, National Chung-Hsing University, Taichung, Taiwan; 4 Department of Neuroscience, Uppsala University, Uppsala, Sweden; 5 Department of Poultry Science, North Carolina State University, Raleigh, North Carolina, United States of America; Florida International University, United States of America

## Abstract

Dermal hyperpigmentation or Fibromelanosis (FM) is one of the few examples of skin pigmentation phenotypes in the chicken, where most other pigmentation variants influence feather color and patterning. The Silkie chicken is the most widespread and well-studied breed displaying this phenotype. The presence of the dominant *FM* allele results in extensive pigmentation of the dermal layer of skin and the majority of internal connective tissue. Here we identify the causal mutation of FM as an inverted duplication and junction of two genomic regions separated by more than 400 kb in wild-type individuals. One of these duplicated regions contains endothelin 3 (*EDN3*), a gene with a known role in promoting melanoblast proliferation. We show that *EDN3* expression is increased in the developing Silkie embryo during the time in which melanoblasts are migrating, and elevated levels of expression are maintained in the adult skin tissue. We have examined four different chicken breeds from both Asia and Europe displaying dermal hyperpigmentation and conclude that the same structural variant underlies this phenotype in all chicken breeds. This complex genomic rearrangement causing a specific monogenic trait in the chicken illustrates how novel mutations with major phenotypic effects have been reused during breed formation in domestic animals.

## Author Summary

The process of animal domestication has been a long and ongoing effort of the human race to cultivate beneficial traits in agriculturally productive or otherwise beneficial species. We are just now beginning to understand the effect this type of selection pressure has had on genetic variation and overall genome architecture using quickly advancing modern genetic and genomic technologies. Here we show how along the path of animal domestication a single large rearrangement involving a duplication and inversion of two distinct regions of the chicken genome occurred, likely disrupting long-range cis-regulatory elements of endothelin 3 (*EDN3*) and resulting in a very extreme skin pigmentation phenotype. Dermal hyperpigmentation, or Fibromelanosis (FM), is a defining characteristic of the Silkie chicken breed, which originates in China. Chickens very similar to the Silkie have been described in ancient Chinese texts on traditional medicine, illustrating how unique phenotypes in domesticated animals are incorporated into human culture and tradition that persists to this day. The presence of the same rearrangement in other FM chicken breeds found around the world highlights both the causality of this mutation as well as how humans serve to spread genetic variation linked to novel traits in domestic animals.

## Introduction

**Figure 1:**
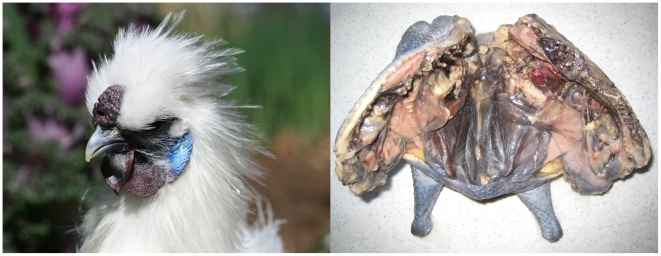
The Silkie chicken displaying the Fibromelanosis phenotype. An adult White Silkie Bantam chicken (left). Hyperpigmentation of the comb, wattle, face, and beak is clearly visible. A White Silkie Bantam (right) prepared in a typical manner for meat consumption, having being split down the spine with viscera removed. Intense pigmentation of internal connective tissue and the exterior skin is evident while muscle tissue remains normally pigmented.

Fibromelanosis (FM) is characterized by intense pigmentation of the dermal layer of skin across the entire body, which results in a dark blue appearance when viewed through the clear epidermis (Figure 1). The term Fibromelanosis was coined to denote the association of pigmentation with internal connective tissue [1] and can be readily seen in the trachea, pericardium, blood vessels, sheaths of muscles and nerves, gonads, mesenteries of the gut, and periosteum of bone [1]–[5].

The Silkie breed is the most widespread and well-studied breed displaying FM. Silkie chickens present a unique collection of interesting phenotypes; the namesake Silkie feathering trait, blue earlobes, polydactyly, walnut comb, crest, beard, vulture hock, and feathered legs, all of which may have contributed to the human fascination and subsequent global distribution of this breed seen today [1], [4], [6]. Silkies are very popular with exhibition and backyard poultry breeders in the USA and Europe and are also available in many Asian grocery stores within the USA. The Fibromelanosis (FM) or dermal hyperpigmentation phenotype of the Silkie chicken is one of only a few skin pigmentation mutants in the chicken and has been a subject of cultural importance and scientific interest for centuries. This breed is thought to originate in China and closely resembles fowl described in 16th century Chinese texts on traditional medicine, although the exact origin of the Silkie breed is unknown [7]. Marco Polo's description of chickens that “have hair like cats, are black, and lay the best of eggs” in 1298 or Aldrovandi's account of “wool-bearing” chickens with white feathers and five toes in 1600 may refer to the Silkie [8], [9], and there are numerous vague references to chickens with similar features to the Silkie in much older Chinese texts. Indeed, folklore describes the Silkie chicken as receiving healing properties after eating pills of immortality created by the deity Lu Dongbin at Tiger-Nose peak. Although the most common globally, the Silkie chicken is not the only breed with dermal hyperpigmentation. Other FM strains are found in India, Indonesia, Japan, Korea, Sweden and Vietnam with varying degrees of overall phenotypic similarity to the Silkie (personal observations).

One of the earliest studies of the Silkie dermal hyperpigmentation phenotype was by Bateson and Punnett in 1911 [10] which together with the work of Dunn and Jull [11] showed the autosomal dominant nature of the *FM allele in conjunction with the sex-linked *Inhibitor of Dermal Melanin* (*ID*) locus acting upstream of *FM; here we have adopted the currently recommended nomenclature system for the chicken where *FM* refers to the *Fibromelanosis* locus and *FM and *N refer to the dominant Fibromelanosis inducing allele and the recessive normally pigmented wild-type allele respectively. All birds expressing the FM phenotype are homozygous wild-type *N at the *ID* locus, or hemizygous in the case of females as *ID* is located on the Z chromosome.

Melanocytes are derived from neural crest cells (NCCs), a multi-potent population of cells emigrating from the dorsal neural tube. In the Silkie embryo melanoblasts, the NCC-derived precursors of pigment-producing melanocytes, enter a migratory pathway that is normally reserved for NCCs of the neuronal and glial cell lineages [12]. This results in colonization of target tissues which normally are not exposed to melanoblasts and would otherwise remain unpigmented. In addition to this abnormal choice of migratory pathway melanoblasts also accumulate in large numbers throughout the body plan of the Silkie embryo [2]. This suggests a two-fold molecular mechanism of ectopic migration and continued proliferation, which may correspond to the classically described *ID* and *FM* loci, respectively. Previous embryo grafting experiments have clearly shown the proliferative effect of the Silkie tissue environment on melanocyte behavior but have been unable to determine if Silkie melanocytes possess inherent differences in migratory ability or if this also is a non-cell autonomous attribute of the Silkie [13].

Here we show that FM is caused by an inverted duplication of two genomic regions, each greater than 100 kb, located on *Gallus gallus* autosome 20, which results in increased expression of endothelin 3 (*EDN3*).

## Results

### Identification of the genomic region associated with *FM*

Using a backcross mapping population we previously identified a 2.8 Mb region of chromosome 20 that was completely associated with the dermal hyperpigmentation phenotype corresponding to the *FM* locus [6]. Using this same mapping population and additional markers we have now refined this region to 483 kb (10,518,217–11,000,943 bp) of chromosome 20 which is completely associated with FM in 270 backcross individuals; all genome coordinates are respective to the May 2006 (WUGSC 2.1/galGal3) assembly [14]. Identity by descent analysis in a diverse panel of chicken breeds identified a 75 kb haplotype (10,717,600–10,792,608 bp) within this 483 kb region which contained five SNPs observed to be heterozygous in all *FM samples (Figure S1). Two of these five SNPs (rs16172722 and rs16172768) were fixed for the reference allele in the diverse breed panel wild-type individuals. The other three SNPs (GGaluGA180596, rs16172794 and rs16172818) were segregating for both alleles to various degrees in wild-type individuals.

### Identification and characterization of a complex structural rearrangement showing complete concordance with *FM*

**Figure 2:**
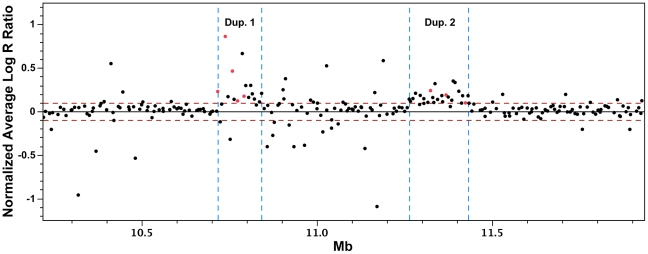
Group-wise analysis of Log R ratio SNP data for the detection of copy number variations. Log R ratio data from wild-type and FM individuals were partitioned based on *FM* genotype. The average of the wild-type group was subtracted from the average of the *FM group on an individual SNP basis and plotted by genomic base pair coordinate. Two distinct genomic regions with elevated Log R ratio values are evident (red dotted line = ±0.1). SNPs marked in red were always observed in the heterozygous state in FM individuals in both duplicated regions.

**Figure 3:**
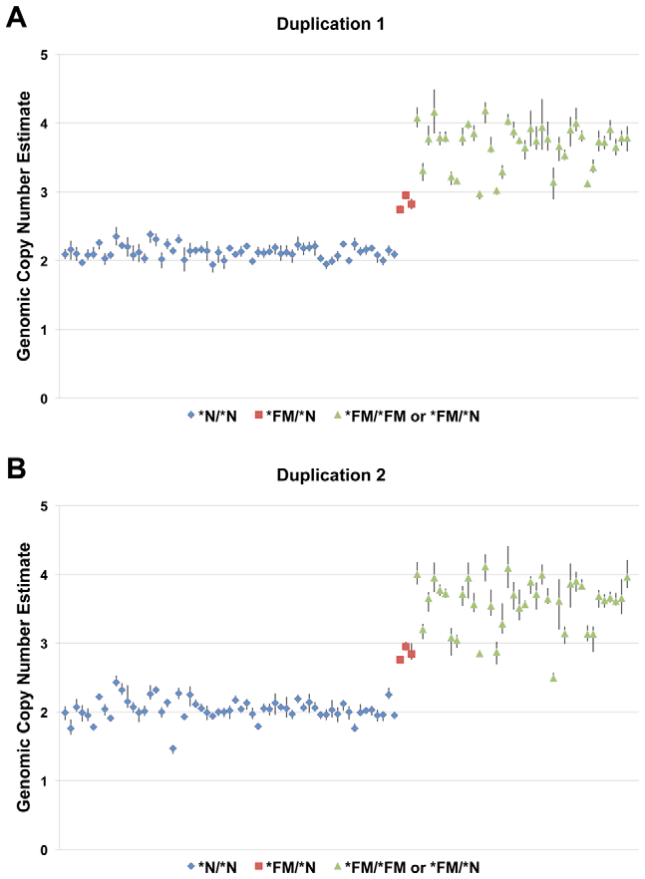
A two-fold increase in genomic copy number is associated with FM. Genomic copy number was estimated using qPCR for a large panel of individuals with known skin pigmentation status. Panel A depicts a primer/probe set located within the first duplicated region and panel B depicts a primer/probe set located within the second duplication. Three known heterozygotes (red) show an estimated copy number of approximately 3 as compared to wild-type individuals (blue) with a copy number of 2. Individuals known to carry at least one *FM allele (green) cluster towards an estimated copy number of four, although it is evident that some heterozygotes are likely included in this group.

The observation of fixed heterozygosity in *FM individuals prompted an analysis of copy number variation using the 60K Chicken iSelect chip Log R ratio data from the diverse breed panel. The GenePattern implementation of the Circular Binary Segmentation (CBS) algorithm [15], [16] identified a region with elevated Log R ratio levels in *FM individuals indicative of a duplication, although not all *FM individuals surpassed the significance threshold (Table 1). This region overlapped the five previously described heterozygous SNPs. This analysis also suggested a second putative duplication event at 11.1–11.4 Mb on chromosome 20 in *FM individuals. To further define the exact boundaries of the putative duplications we performed a group-wise analysis by subtracting the average Log R ratio of known *N individuals from the average of *FM individuals on a single SNP basis. This method revealed a clearer picture of the two putative duplicated regions in *FM individuals from 10,717,600–10,842,919 bp and 11,264,226–11,432,336 bp (Figure 2). Quantitative PCR (qPCR) analysis confirmed the duplication of both genomic regions in FM birds with an estimated copy number of approximately 1.5–2× that of wild-type individuals, indicating that some FM birds were likely heterozygous for a mutant allele composed of a 2× duplication (Figure 3).

**Table 1:** Duplicated genomic regions associated with the FM phenotype identified using SNP data from the 60K Chicken iSelect chip on an individual basis.

Duplication	Bird ID	Breed	Region (bp)	# of Markers	Avg. Log R Ratio
1	102	Ayam Cemani	10,695,372–10,893,420	31	0.18
	1746	Silkie	10,714,506–10,906,537	30	0.18
	1747	Silkie	10,703,086–10,906,537	31	0.17
	1748	Silkie	10,714,506–10,906,537	30	0.16
	G10	Silkie	10,728,799–10,850,224	18	0.27
2	1746	Silkie	11,166,005–11,432,336	40	0.14
	1747	Silkie	11,166,005–11,432,336	40	0.12
	G10	Silkie	11,166,005–11,435,087	41	0.18

**Figure 4:**
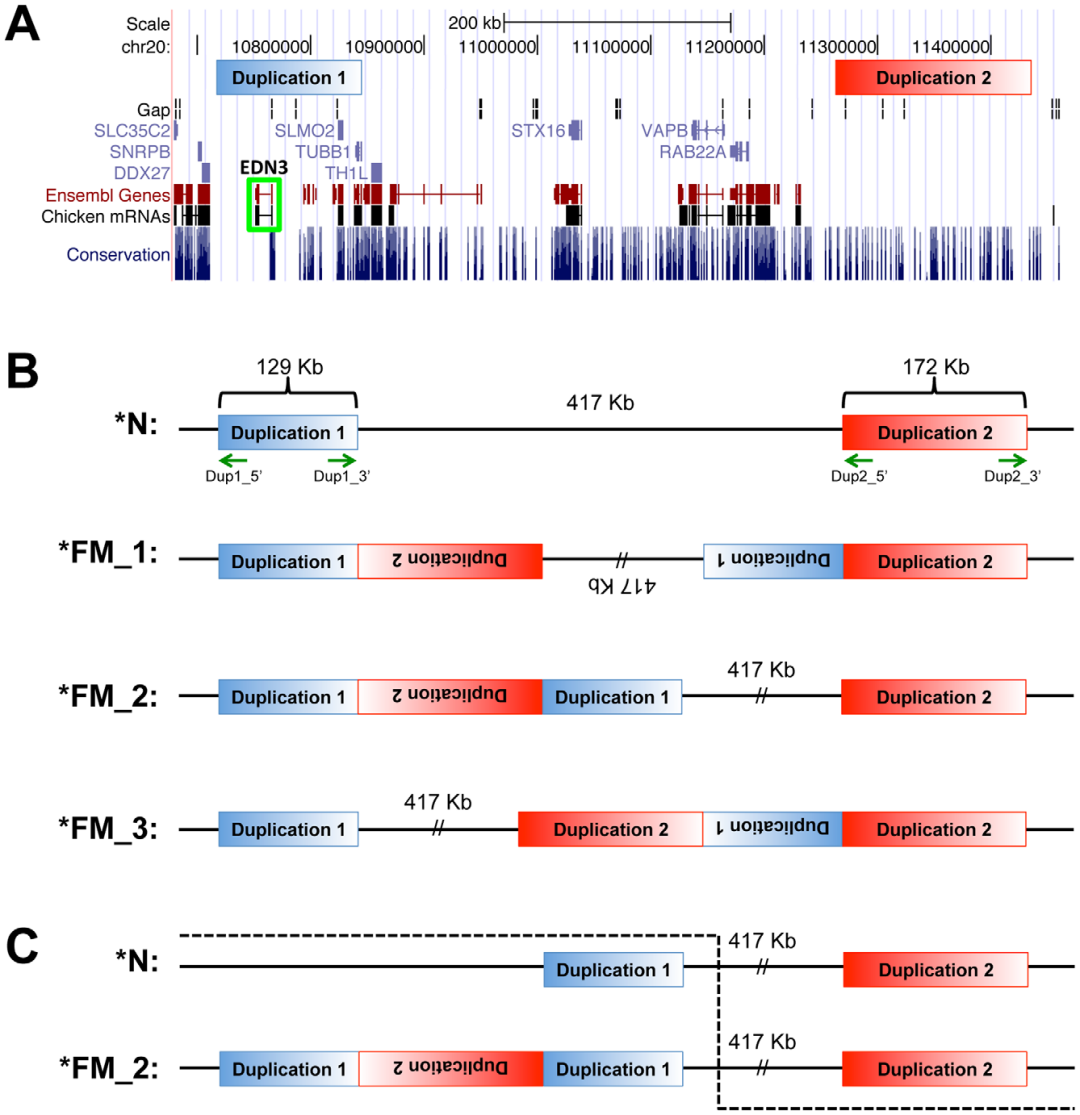
Genome view of duplicated regions and possible rearrangement scenarios. (A) The location of the two duplicated regions is depicted in blue and red respectively. The location of *EDN3* is outlined in green. Several other genes are located within the first duplicated region while no known coding elements are found within the second duplicated region. Image was generated with the UCSC Genome Browser (http://genome.ucsc.edu) using the May 2006 (WUGSC 2.1/galGal3) assembly. (B) The structural arrangement of the *FM* locus was tested with outward facing primers (green arrows) at the boundary of each duplicated region. The amplification pattern obtained using different primer combinations was consistent with three different rearrangement scenarios; *FM_1, *FM_2, and *FM_3 as compared to the wild-type *N arrangement. (C) A single individual in the backcross mapping population strongly supports the *FM_2 rearrangement scenario. This individual was a recombinant in the 417 kb single copy region as represented by the dashed line, possessing the alleles inherited from the *N founder prior to the crossover event and the alleles inherited from the *FM founder from the crossover onwards. This individual was phenotypically wild-type and had the normal copy number of both duplicated regions, all of which supports *FM_2 as the only possible arrangement of the *FM allele.

Although the second duplicated region lies outside the 483 kb region we had identified in the mapping population, the presence of both duplicated regions in all *FM individuals from the diverse breed panel suggested that both regions were involved in a genomic rearrangement and duplication event associated with the *FM* locus (Figure 4A). We investigated the structural arrangement of the putative duplicated regions by PCR between outward facing primers at each end of both putative duplicated regions. We first tested for the presence of a tandem duplication using primers Dup1_5'xDup1_3' and Dup2_5'xDup2_3', however no amplification was detected. After testing all possible combinations of these four primers successful amplification was detected only for Dup1_5'xDup2_5' and Dup1_3'xDup2_3', suggesting that each duplicated region was joined to the other in an inverted orientation (Figure 4B). Sequencing of these PCR products revealed the exact coordinates of the first duplicated region to be 10,717,294–10,846,232 bp and the coordinates of the second duplicated region to be 11,262,904–11,435,256 bp. In conjunction with the successful amplification of the two PCR products described above, amplicons were successfully generated across the wild-type boundaries of both duplicated regions in several known *FM homozygotes. A three primer diagnostic test for each duplicated region was developed that is capable of amplifying both a wild-type and mutant allele in the same reaction (Table 2 and Figure S2). All samples from the diverse breed panel and other populations known to carry *FM tested positive for both duplicated region junctions while no *N individuals were found to carry either of these rearrangements (Table 3). In order to differentiate *FM/*FM from *FM/*N individuals a qPCR genomic copy number assay must be used due to the retention of all four duplication boundary wild-type sequences in the mutant allele.

**Table 2:** Diagnostic test for the duplication and rearrangement associated with the FM phenotype in chickens.

Assay	Primer 1	Primer 2	Product Size (bp)	Description
A	232	234	379	Duplication 1 5′ wild-type
	200	234	280	Duplication 1 5′ to Duplication 2 5′ mutant
B	201	202	302	Duplication 2 3′ wild-type
	197	201	159	Duplication 1 3′ to Duplication 2 3′ mutant

See Figure S2 for agarose gel image of PCR products.

See Table S7 for primer sequences.

**Table 3:** A PCR-based diagnostic test reveals complete association of two inversion junctions with the FM phenotype in chickens.

	Genotype	
Breed	*N/*N	*FM/-a
FM		
Ayam Cemani	0	7
Black H'Mong	0	8
Silkie^b^	0	39
Svarthöna	0	4
Known Heterozygote		
Silkie crossbred	0	3
Wild-type		
Ameraucana	1	0
Araucana	4	0
Brahma	4	0
Campine	2	0
Choi	8	0
Cochin	3	0
Crossbred Egg Layer	11	0
Dong Tao	8	0
Dorking	2	0
Faverolle	2	0
Hamburg	4	0
Houdan	2	0
Leghorn	2	0
Orpington	1	0
Plymouth Rock	3	0
Polish	4	0
Red Junglefowl	1	0
Sebright	4	0
Sultan	2	0
Sussex	2	0
Tre	8	0
Wyandotte	4	0

a *FM/- = *FM/N or *FM/FM.

b Silkie samples represent five different sub-lines from China, USA, Sweden, and Vietnam.

Our data suggest three possible spatial arrangements of the duplicated regions, which are indistinguishable from each other via conventional PCR assays across duplication boundaries (Figure 4B). Our data favors rearrangement scenario *FM_2 given the detection of a single recombination event between the duplicated regions in our backcross population. This recombinant individual was phenotypically wild-type and had inherited a Silkie chromosome beginning in the 417 kb single copy region between the two duplications and continuing through the second duplicated region until the end of the chromosome (Figure 4C). This effectively eliminates rearrangement scenario *FM_1 as recombination between inverted copies of the intervening single copy region would result in chromosomal loss. Scenario *FM_3 also can be eliminated given that in this arrangement recombination between the single copy region of the depicted *N and *FM_3 alleles would result in the retention of duplicated copies of both regions, which was not the case in this recombinant individual as estimated by the genomic qPCR assay.

Sequencing of ∼800 bp at each of the duplication boundaries, in their wild-type arrangement, revealed a high degree of sequence variation between and within four different populations of Silkie chickens known to be homozygous *FM (Tables S1, S2, S3, and S4). Conversely, at the junction of the 5′ and 3′ ends of each duplicated region, respectively, there was no sequence variation between any of these same *FM samples (Tables S5 and S6). The absence of sequence variation at both duplication junction points suggests that this haplotype has been highly conserved since the mutation occurred, but that with increasing distance from the duplication junction points recombination has occurred with other alleles. The single recombinant individual observed in the mapping population supports this view.

**Figure 5:**
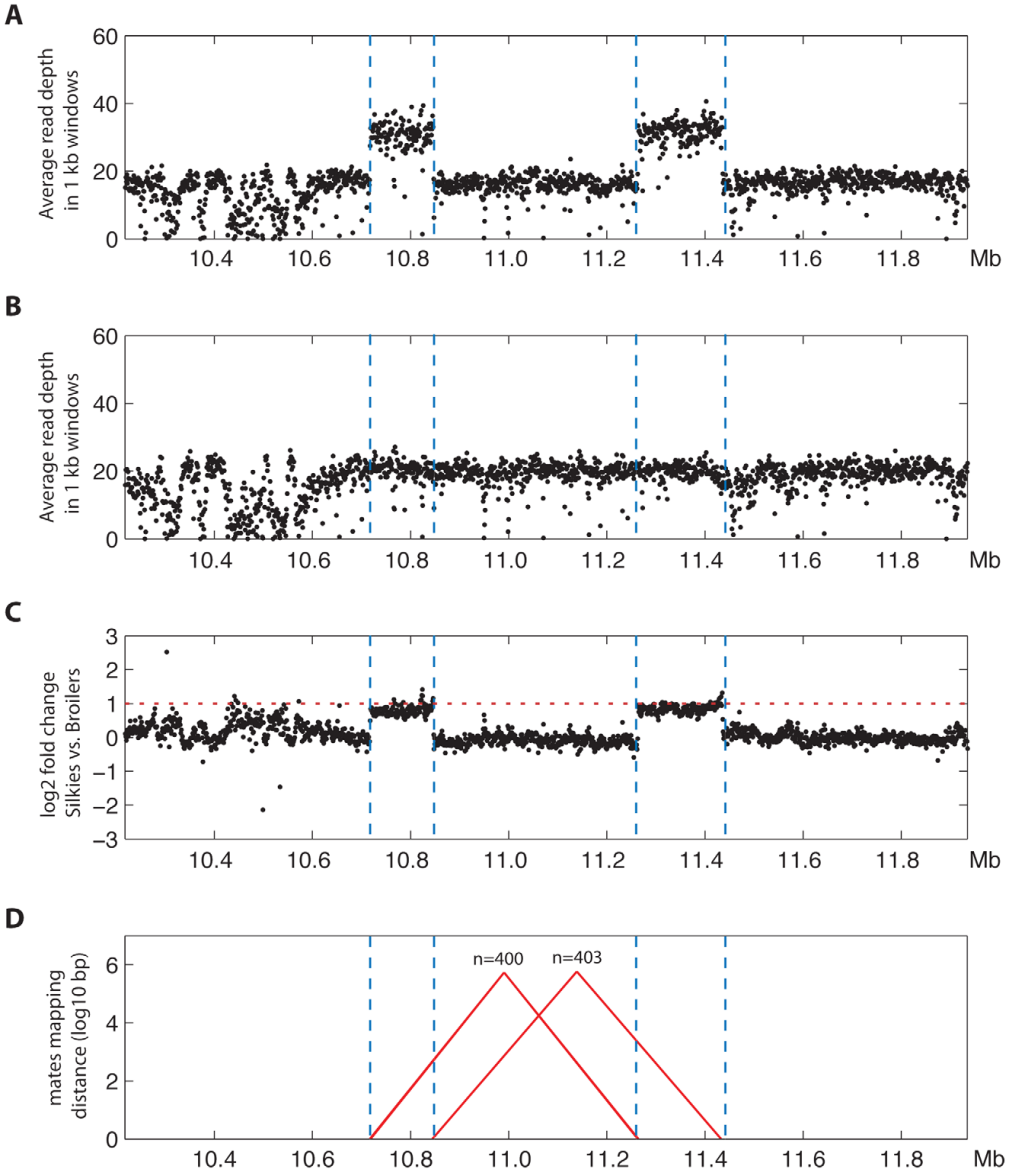
Massively parallel sequencing confirms the inverted duplication corresponding to the FM locus. Average sequencing read depths in windows of 1 kb along the interval 10.218–11.935 Mb on chromosome 20 for (A) the Silkie pool and (B) the Broiler pool. (C) Log2 fold change values (normalized for read depth) between Silkie and Broiler pools showing that the interval within the two large duplications (area between blue vertical dotted lines) has approximately twice as high levels of sequence coverage in the Silkie pool than in the Broiler pool (2× higher levels indicated by the horizontal red dotted line). (D) The mate-pair information was used to plot all candidate structural variants in the region of interest. Candidate structural variants were defined as windows where at least 20% of the mate pairs had mapping distances exceeding six standard deviations above the average mapping distance for chromosome 20 and had mapping distances ranging ±1500 base pairs from the median distance observed for those exceeding 6 standard deviations. On the y-axis the size of candidate structural variants are presented in log10 base pairs and the x coordinates of connected colored lines indicate the genomic coordinates of the pairs supporting structural variants (red = mate-pairs map to different strands, which is indicative of an inversion). The number of mate-pairs supporting a feature is indicated above the feature.

We decided to take advantage of massively parallel whole genome sequencing data to verify the duplicated regions and their orientation. We compared the read depth between FM and wild-type DNA pools, 15–20 birds in each pool, sequenced on average to 30× coverage. In the FM pool there was approximately 2-fold higher coverage strictly within the duplicated regions we had previously identified (Figure 5A–5C). A large number of mate-pair sequencing reads were detected that confirmed the inverted arrangement of the duplicated regions; one set of approximately 400 mate-pairs mapped to the 5′ area of each duplicated region and a second similar number of mate-pairs mapped to the 3′ area of each duplicated region. No other duplications, deletions or rearrangements in this genomic region were detected in this analysis (Figure 5D).

### *FM* is associated with increased *EDN3* expression in embryonic and adult tissue

**Figure 6:**
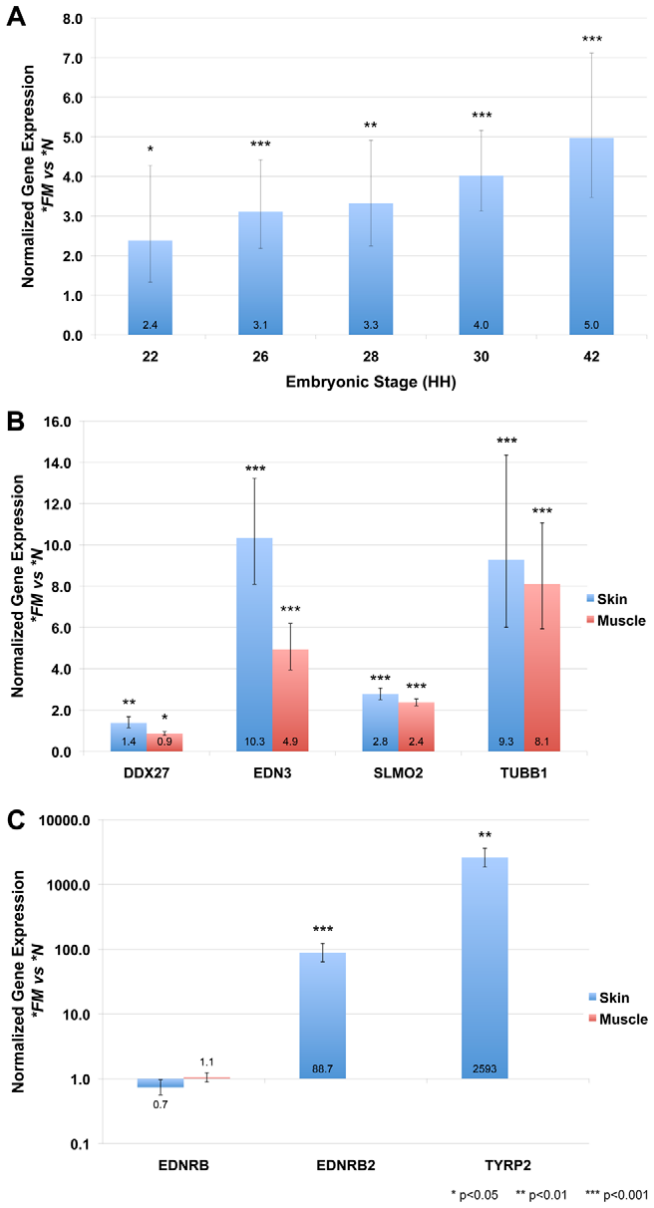
Genomic rearrangement drives increased expression of genes located within the duplicated region in FM embryonic and adult tissues. Gene expression analysis by SYBR Green qPCR of *FM (Silkie breed) tissue normalized to the *N (New Hampshire breed) and calibrated to glyceraldehyde 3-phosphate dehydrogenase (*GAPDH*). Error bars indicate 95% confidence intervals and significance thresholds are indicated. (A) Embryo tissue cross sections were collected at the level of the wing bud at the indicated stages. *EDN3* is upregulated at all developmental stages assayed, with an increasing magnitude of differential expression in *FM tissue with age. (B) Genes located within the first duplicated region (*EDN3*, *SLMO2* and *TUBB1*) are significantly increased in expression in adult skin and muscle tissue of *FM chickens. The expression of *DDX27*, located adjacent to but outside of the duplicated region, is also significantly differentially expressed, but does not show the same degree of upregulation as the genes within the duplication. (C) The expression of the EDN3 receptor *EDNRB* is not significantly different in *FM skin or muscle tissue when compared to *N tissue. However, the expression of the EDN3 receptor *EDNRB2*, expressed predominately by melanocytes, is highly upregulated in *FM skin tissue. A key component of the melanin biosynthesis pathway, *TYRP2*, is also highly upregulated in *FM skin tissue. *EDNRB2* and *TYRP2* expression was not detected in *N muscle tissue, so no comparison can be made to *FM skin tissue.

There are several known coding elements within the first duplicated region including *ATP5E* (ATP synthase epsilon subunit), *TUBB1* (tubulin, beta 1), *SLMO2* (slowmo homolog 2) and *EDN3* (endothelin 3). EDN3 has a known role in melanocyte regulation [17]–[19] and was an obvious candidate gene for further analysis. The second duplicated region does not contain any known coding or regulatory elements but displays isolated pockets of elevated conservation scores across seven vertebrate species as calculated using phastCons [20] in the UCSC Genome Browser (http://genome.ucsc.edu) (Figure 4A). The entire coding sequence of *EDN3* lies approximately in the center of the first duplicated region. In the FM (Silkie breed) embryo *EDN3* is significantly (p<0.05) increased in expression during embryonic stages when melanoblasts are migrating and beginning to differentiate into melanocytes (Figure 6A). The magnitude of increased expression of *EDN3* in FM embryos appears to increase with developmental age and reaches a remarkably high level of differential expression (about 10-fold) in adult skin tissue (Figure 6B). The expression of two other genes, *SLMO2* and *TUBB1*, located within the first duplicated region are also significantly increased in expression in both skin and muscle tissue from adult FM chickens (Figure 6B). The expression of *DDX27* (DEAD (Asp-Glu-Ala-Asp) box polypeptide 27), located outside but in close proximity to the first duplication is significantly differentially expressed in FM skin and muscle (up and down, respectively), but the magnitude of the difference is minimal when compared to the genes within the duplication; *EDN3*, *SLMO2* and *TUBB1* (Figure 6B).

We also examined the expression of two EDN3 receptors in adult chicken skin and muscle tissue; *EDNRB* (endothelin receptor B) which in the chicken is confined to non-melanocyte derivatives of the neural crest migrating through the dorsoventral pathway [21] and *EDNRB2* (endothelin receptor B subtype 2) which is expressed primarily by melanocytes and is involved in cell migration and differentiation [22], [23]. The expression of *TYRP2* (tyrosinase-related protein 2), which catalyzes the conversion of L-Dopa into DHICA [24], was also assayed as an indication of the level of eumelanin biosynthesis occurring. In wild-type muscle tissue *EDNRB2* and *TYRP2* expression was below the detection level of the qPCR assay, while no significant difference in *EDNRB* expression was detected between FM and wild-type muscle or skin tissue (Figure 6C). The expression of *EDNRB2* and *TYRP2* was significantly higher in FM skin tissue as compared to wild-type skin (>88 and >2500 fold, respectively) (Figure 6C). Most notably, the expression of *EDNRB2* and *TYRP2* was 26-fold and 5-fold higher, respectively, in wild-type skin tissue as compared to FM muscle tissue (Figure S3). This reflects the absence of pigment producing melanocytes in FM muscle even though *EDN3* expression is upregulated in both FM skin and muscle tissue. Note that while wild-type skin dermis is unpigmented, there are active melanocytes within the feather follicle.

The exonic sequence of the *EDN3* transcript was sequenced from genomic DNA (data not shown), including across a gap in the current genome assembly that corresponds to a portion of the second coding exon. In these samples no non-synonymous sequence variants were detected between FM and wild-type individuals.

## Discussion

We have demonstrated that the FM phenotype shows complete concordance with a complex structural variant involving the duplication of two genomic regions, each larger than 100 kb and separated by 417 kb on wild-type chromosomes. The precise definition of the structural variant was facilitated by our use of a modified Log R ratio analysis of SNP data generated using the Illumina 60K Chicken iSelect chip. By comparing *FM and *N individuals in a group-wise manner, we were able to predict the duplication boundaries to an accuracy of 300–3,300 bp from the actual breakpoints, limited primarily by the spacing of markers on the chip. This novel structural variant was not found in samples of Red Junglefowl (*Gallus gallus*) or in a diverse panel of domestic chickens, representing 21 different breeds all of which do not display the FM character. However, the same complex rearrangement associated with FM was found in four different breeds representing four countries and two continents, Silkie from China, Ayam Cemani from Indonesia, Black H'Mong from Vietnam and Svarthöna from Sweden. The result is consistent with that of a single mutation event underlying FM in all breeds. Sequence analysis of ∼800 bp PCR fragments centered on all four duplication boundaries and both duplication junctions revealed substantial sequence polymorphisms between and also within *FM populations at the wild-type duplication boundaries, but complete sequence conservation at both duplication junctions. This supports the causal nature of the inverted duplications for the FM phenotype and suggests that the mutant allele has experienced recombination with wild-type alleles with increasing distance from the inverted duplication junction points.

**Figure 7:**
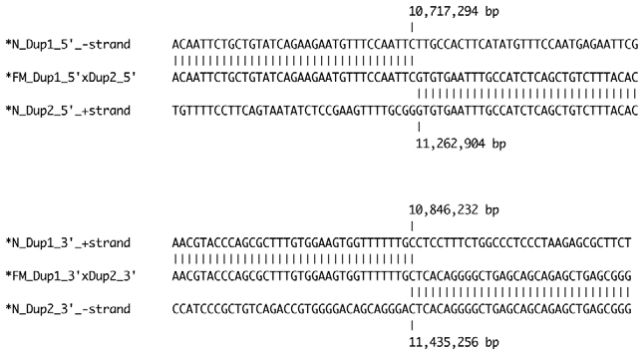
Sequence alignment of duplication junction points. The junction of the 5′ portion of the duplicated regions was detected using primers Dup1_5' and Dup2_5' and the junction of the 3′ portion of each duplicated region was detected using primers Dup1_3' and Dup2_3'. No sequence homology, insertion or deletion was detected at the breakpoints except for the overlap of a single C nucleotide at the junction of the 3′ portion of the duplicated regions.

We have demonstrated that the structural variant underlying FM involves the joining of the 5′ ends of the two duplicated regions as well as the joining of the 3′ ends of the two duplicated regions, while still allowing for the successful PCR amplification of all four wild-type duplication boundary sequences. We searched for homologous sequences at the duplication junction boundaries in order to infer the mechanism of this genomic rearrangement event. We detected only a single base pair of homology at the immediate junction of the 3′ ends of both duplicated regions while no homology was detected at the junction of the 5′ ends of both duplicated regions (Figure 7). This lack of sequence homology suggests a mechanism such as non-homologous end joining (NHEJ) via double-strand break repair. However the complex nature of this rearrangement with two distinct duplicated regions joined in an inverted fashion with no overall gain or loss of sequence at the junction points is difficult to reconcile with the known mechanisms of NHEJ in which sequence is often added or deleted at the breakpoint [25], [26]. Alternative mechanisms, which rely on a small amount of sequence homology, are fork stalling and template switching (FoSTeS) and micro-homology mediated break induced replication (MMBIR) [27], [28]. The FoSTeS/MMBIR DNA replication based mechanisms have been proposed for many complex rearrangements similar to the one we have identified as causing FM but typically relies on 2–5 bp of micro-homology at the junction point, although examples relying on a single base pair have been described [27]. No previously annotated segmental duplications were found in proximity to any of the four duplication boundaries [29]. We cannot exclude the possibility of additional genetic elements being involved in the formation and current structure of this genomic rearrangement but the analysis of massively parallel sequencing data from the FM DNA pool suggests that we have identified all major aspects of this genomic rearrangement (Figure 5).

A characteristic feature of duplicated sequences is that they are prone to copy number variation due to unequal crossing-over. This is well illustrated by the dominant white locus in pigs that shows extensive haplotype diversity in breeds with the dominant white color based on the presence of 1–3 copies of a 450 kb duplication encompassing the entire *KIT* gene [30]. The FM rearrangement appears to be stable, unequal crossing-over is probably suppressed by the presence of an inverted copy of Duplication 2 located between the two copies of Duplication 1 (Figure 4). Our whole genome resequencing of pooled samples and qPCR analysis of individual samples did not indicate the presence of FM chromosomes with more than two copies of the duplicated sequences (Figure 3 and Figure 5).

We have shown that the structural variant underlying FM is associated with an increased expression of endothelin 3 (*EDN3*), located completely within the first duplicated region (Figure 4A). EDN3 has been shown to have a mitogenic effect on melanocytes *in vitro* [17]–[19], which is similar to the proliferative effect extracts from Silkie embryos have on NCCs [31]. Furthermore, a transgenic mouse model of ectopic *EDN3* expression results in dermal hyperpigmentation [32], [33] that is strikingly similar to that of the Silkie chicken. In chicken wild-type embryos, NCCs specified as melanoblasts begin to delaminate from the dorsal neural tube at stage 18 and migrate almost exclusively within the dorsolateral pathway. In the Silkie embryo the timing of melanoblast migration from the neural crest is slightly delayed but perhaps more interesting is the transit of melanoblasts through both the dorsoventral and dorsolateral migratory pathways. In addition to this abnormal choice of migratory pathway, melanoblasts appear to continue to proliferate in the Silkie embryo giving rise to a large number of pigmented melanocytes in ectopic positions of the embryo already at embryonic day 18 [2]. We show that in the developing embryo, when melanoblasts are in the process of migrating to their target tissues, there is already increased expression of *EDN3* in the Silkie embryo that continues to increase in the magnitude of differential expression throughout development. It is quite remarkable that in the adult skin tissue of Silkie chickens *EDN3* expression is increased about 10-fold (Figure 6B). This suggests a possible role for EDN3 in melanoblast maintenance later in life given that continued expression of *EDN3* in a transgenic mouse model was necessary for maintenance of the dermal hyperpigmentation phenotype after birth [33]. The expression of *EDN3* was significantly increased in both FM skin and muscle tissue, but the expression of genes indicative of the presence of active melanocytes was lower in FM muscle than in wild-type skin tissue. This suggests that while *EDN3* may be upregulated in both FM skin and muscle, few melanocytes in the muscle are present and/or able to respond and thus FM muscle remains largely unpigmented.

The *FM* locus constitutes both a cis-acting and trans-acting eQTL. For instance, we observed a staggering >2500-fold upregulated expression of *TYRP2*, a key gene in the melanin biosynthesis pathway, in FM skin compared with wild-type skin (Figure 6C). This is partially explained by the dramatic increase in melanocyte numbers in FM skin and partially by downstream effects of increased EDN3 signaling on these melanocytes. Thus, a whole transcriptome comparison of FM and wild-type skin is expected to reveal significant differential expression of hundreds, if not thousands, of transcripts and it would be challenging to reveal the causative nature of these alterations without a genetic analysis like the present study.

Previous experiments documenting the migration pattern of melanoblasts in the Silkie embryo utilized the White Leghorn and Light Brown Leghorn as the control breed [2], [34]. Unfortunately, both of these control breeds possess opposing alleles at both the *FM* and *ID* (*Inhibitor of Dermal Melanin*) loci compared to the Silkie. This complicates the reconciliation of increased *EDN3* signaling with the abnormal migration pattern and increase of melanoblasts previously documented in the Silkie, as these developmental differences could be due to either *ID* or *FM*. We have performed *in situ* hybridization analysis of *EDN3* expression (data not shown) and did not observe any difference in spatial expression when compared to a FM*N control breed carrying the same *ID* allele (*N) as the Silkie. The elevated level of *EDN3* expression detected via qPCR, but without any detectable difference in *EDN3* spatial expression pattern from the *in situs*, is consistent with the perspective of *ID* controlling pathways of melanoblast migration and *FM* being responsible for melanoblast proliferation and maintenance. Even in the presence of *FM and the associated increased expression of *EDN3*, individuals carrying the ID*ID allele lack visible pigmentation except for a small number of isolated patches most easily visible on the shank. In FM*N ID*N chickens pigmentation of the skin is only seen in the dermis of the shank, which could be explained by extensive migration of melanoblasts throughout the body plan as a result of ID*N, but lack of melanoblast maintenance and proliferation due to FM*N except for in the shank where expression of *EDN3* may be under different regulatory influences. Further work directed at identifying the *ID* causal mutation located on the Z chromosome is necessary to further explore this hypothesis.

The FM structural rearrangement results in the duplication of a key regulator of melanoblast and melanocyte proliferation and maintenance, *EDN3*. Loss of function mutations in *EDN3* are associated with pigmentation, auditory and gut innervation disorders in humans (Waardenburg Syndrome and Hirschsprung Disease) [35] and in the mouse (lethal spotting) [36] suggesting a crucial role for EDN3 in regulating NCC-derived lineages. The long standing view on the origin of melanocytes in all tissues except the retinal pigmented epithelium is that they are derived from NCCs which are fate specified as melanoblasts and exploit the dorsolateral migratory pathway [37]. However, recent evidence has shown that in the mouse and chicken melanocytes derived from Schwann cell precursors, which originally migrated through the dorsoventral pathway, make an equivalent contribution to mature melanocytes as do melanoblasts which have migrated through the canonical dorsolateral pathway [38]. In light of how EDN3 promotes the reversion of melanocytes and glia to a bipotent precursor [17], [18], [39] it is interesting to speculate on how increased EDN3 signaling may affect the relative contribution these two sources of melanocytes make in FM chickens. FM chickens do not appear to suffer from any gross neurological defects suggesting that increased EDN3 signaling does not have a major impact on the development of the completely neural crest-derived peripheral nervous system in this strain.

There are several different varieties of the Silkie chicken breed as distinguished by feather color (white, buff, red, blue, grey, partridge, and black), which indicates that *FM* does not affect feather pigmentation. Pigmentation of the feather is controlled by the activity of epidermal melanocytes and therefore this suggests non-overlapping mechanisms regulating the development of dermal and epidermal melanocytes. In the mouse ectopic expression of *EDN3* results in hyperpigmentation of the dermis by melanocytes that are more similar to non-cutaneous melanocytes of the eye, inner ear, and harderian gland than they are to epidermal melanocytes in regards to responsiveness to EDN3, hepatocyte growth factor (HGF), and tyrosine kinase (KIT) ligand signaling [32]. These non-cutaneous like dermal melanocytes are incapable of contributing to epidermal hair follicle pigmentation further highlighting the functional differences between these two melanocyte populations [40]. This suggests a strong role for EDN3 in regulating non-cutaneous melanocytes and presents the Silkie chicken as a readily accessible naturally occurring mutant in which to further study this newly described population of melanocytes.

These results provide an example of how a complex structural variation involving an inversion and duplication of two distinct genomic regions each greater than 100 kb can drive a monogenic trait in a domestic animal species. The specific cause of the increased *EDN3* expression remains unclear given the complex nature and size of this structural variant. It is unlikely to be a simple dosage effect given the dominant nature of this mutation as well as our observation of a 10-fold increase in expression of *EDN3* in adult Silkie chicken skin tissue. We propose that the most likely explanation is an altered constellation of long-range cis-regulatory elements affecting *EDN3* expression, disruption of silencer activities and/or recruitment of new enhancer(s) via the physical reorganization of the locus. The observation of increased expression of two other genes within the first duplicated region is consistent with this view of a large-scale perturbation of transcriptional regulation caused by this genomic rearrangement. The increased expression of *SLMO2*, *TUBB1*, and possibly other genes within the duplicated region(s) raises the possibility that multiple genes within this locus are contributing to the dermal hyperpigmentation phenotype, or possibly other more subtle phenotypes not previously associated with FM. We cannot exclude this possibility, but based on the similar dermal pigmentation phenotype seen when *EDN3* is ectopically expressed in the mouse [32], [33] we would suggest that *EDN3* upregulation is the primary driver of dermal hyperpigmentation in FM chickens. To the best of our knowledge there are no other phenotypes consistently observed in all FM breeds, however other researchers in possession of populations segregating at the *FM* locus should explore the possible effect of upregulated expression of *SLMO2* and *TUBB1* on other types of phenotypic variation. It is possible that one of the duplicated copies of *EDN3* has accumulated a gain of function mutation in a regulatory element within the duplicated region, but this appears unlikely since this complex rearrangement was most likely selected by humans due to a striking phenotypic effect associated directly with the inversion and duplication mutation itself.

Several examples of copy number variation linked to single gene traits have previously been described in the chicken [41], [42] and together with the structural variant we have now described as causing FM suggests that structural changes have contributed significantly to the evolution of phenotypic diversity in domestic chickens. Recently the amount of structural variation present in the chicken genome was surveyed using massively parallel sequencing techniques [43], [44], however FM breeds were not included in these analyses. As the technology available to assay genomic structural variation improves through longer sequencing read lengths and the development of high throughput optical mapping [45] there will no doubt be many more cases in which genomic rearrangements are found in association with both monogenic and polygenic traits in many species. As these results have shown, this type of large-scale structural rearrangement of the genome has the capability to alter gene expression regulatory mechanisms at an extended range. This suggests that while this type of variation is inherited as a single locus it may have multiple downstream effects through perturbation of several different genetic pathways, possibly contributing to more subtle or complex phenotypes.

## Materials and Methods

### Animal material for genotyping

Fine mapping of *FM* was performed using a backcross population of chickens as previously described [6]. Silkie chicken breed DNA samples were obtained from several different populations from the USA (North Carolina and Wisconsin), Sweden, Vietnam, and China. These Silkie sub-lines displayed subtle differences in minor breed characteristics reflective of local preferences but overall were representative of the Silkie breed. Samples obtained from other breeds known to display Fibromelanosis included a population of Ayam Cemani, Black H'Mong, and Svarthöna chickens. The Ayam Cemani is an Indonesian breed although the samples used in this study were collected in the USA. The Black H'Mong chicken breed is from the Ha Giang region of Vietnam. The Svarthöna (full name Bohuslän-Dals Svarthöna) is a Swedish breed believed to have been imported from Norway in the early 1900s. A panel of diverse chicken breeds known to be *FM* *N was collected from across North Carolina and Vietnam.

### SNP genotyping

A custom GoldenGate BeadXpress panel (Illumina) containing 36 SNPs spanning the 2.8 Mb region we previously reported as being highly associated with Fibromelanosis [6] was used for fine mapping in the backcross population. The 60K Chicken iSelect chip [46] (Illumina) was used to screen the founders of this backcross population in order to identify completely informative SNPs and was also used to genotype the diverse breed panel and USA Silkie and Ayam Cemani samples for identity by descent haplotype analysis.

### Genomic copy number analysis

The GenomeStudio V2010.3 software package (Illumina) was used to obtain normalized total signal intensity, the Log R ratio, for all SNPs on the 60K Chicken iSelect chip according to the manufacturer's instructions and Peiffer et al. [47]. A continuous region of SNPs having a Log R ratio above/below zero indicates a possible duplication/deletion event. To identify such segments, the exported Log R ratio data from all individuals was analyzed using the Circular Binary Segmentation algorithm implemented in GenePattern [15], [16]. Default parameters were used in the segmentation analysis, i.e. number of permutations used for p-value computation was 10,000, significance level (alpha) for the test to accept change-points was 0.01 and the seed for the random number generator was 12345678. The group-wise Log R ratio was calculated by subtracting the average Log R ratio from all *FM* *N individuals from the average of all *FM* *FM individuals.

TaqMan primer and probe sets were designed using Primer3Plus [48] according to standard parameters. Target probes were 5′ labeled with 6-FAM and 3′ labeled with the minor groove binder (MGB) non-fluorescent quencher, the reference probe located in an exon of *SOX5* was 5′ labeled with VIC and 3′ labeled with TAMRA (ABI). See Table S7 for primer sequences. Reactions were performed using Gene Expression MasterMix (ABI) containing 10 ng of genomic DNA, 800 nM of each primer, 250 nM probe in a total volume of 20 µl. Quality control of all primer/probe sets was evaluated in both simplex and duplex with the control primer/probe set using a 7-point standard curve with a 5× dilution factor and criteria previously described by Ishii et al. [49]: slope −3.1035 to −3.7762 (PCR Efficiency = 92–105%), R>0.995, and y-intercept values differ by less than 1. Genomic qPCR assays were performed using the diverse breed panel, USA Silkie, Chinese Silkie, Svarthöna and Ayam Cemani breed samples as well as several known heterozygotes from the mapping population. Data was analyzed using CopyCaller software (ABI), which uses a ΔΔCt method to first normalize the target Ct value to the reference Ct value within sample, and subsequently normalize all samples to a known calibrator sample. The calibrator sample in this experiment was the wild-type founder of the mapping population, with an expected diploid copy number of 2 for each TaqMan qPCR amplicon. Error bars represent the minimum and maximum estimated copy number as calculated from technical replicates of each sample.

### Generation and analysis of massively parallel sequencing data

DNA from 15 Silkie and 20 commercial Broiler chickens were pooled, resulting in one pool for each breed. SOLiD mate-pair sequencing libraries were generated from these DNA pools with a mean insert size of approximately 2.5 kb. The libraries were sequenced using a SOLiD v.4 instrument (Life Technologies, Carlsbad, U.S.A.) according to the manufacturer's instructions, The reads were mapped to the chicken genome (WUGSC 2.1/galGal3) reference assembly using the Lifescope software (Life Technologies), resulting in average read depths of approximately 30× per library over the chicken genome. The mapping data were used to determine read depths in 1 kb windows for the two samples over the region of interest (10.218–11.935 Mb on chromosome 20). The mapping distances between mate-pairs were used to detect structural variation in relation to the reference assembly.

### Diagnostic test PCR

Outward facing primer sets from each of the four duplication boundary regions were used to determine the spatial arrangement and orientation of the two duplicated regions via PCR. This facilitated the design of primers suitable for sequencing each of the four duplication boundaries and the development of a three primer diagnostic test for the presence of each of the duplications. See Table S7 for primer sequences. The KAPA2G Robust HotStart PCR system (Kapa Biosystems) was used for all standard PCR, the specific parameters of the breakpoint diagnostic test are 1× KAPA2G GC Buffer, 0.2 mM dNTPs, 1.5 mM MgCl_2_, 200 nM of each of the three primers, 0.8 U of KAPA2G Robust HotStart DNA Polymerase, and 50 ng of DNA in a total volume of 20 µl. A touchdown thermal cycling protocol was used for the diagnostic test of 95°C for 5 min, 16 cycles of 95°C, 68°C (−1.0°C/cycle), and 72°C for 30 s each, followed by 24 cycles of 95°C, 52°C, and 72°C for 30 s each.

### RNA isolation and mRNA quantitative PCR

Tissue was collected from Silkie (*FM) and New Hampshire (*N) breed embryos at the level of the wing bud at stages 22, 26, 28, 30, and 42 according to Hamburger and Hamilton guidelines [50] as well as from adult skin and muscle tissue. At least three biological replicates of each breed were collected at all time points. Tissue was homogenized in Mini-Beadbeater (BioSpec) tubes containing TriZol and 1.0 mm glass beads. RNA isolation was performed using the PureLink Micro-Midi kit with TRIzol (Invitrogen) and included an on column DNase treatment.

GenBank accession numbers and associated primer sequences for all genes can be found in Table S8. Reactions were performed using iScript One-Step RT-PCR Kit With SYBR Green (Bio-Rad) containing 50 ng of RNA and 600 nM of each primer in a total volume of 25 µl. Thermal cycling parameters were as described by the manufacturer. PCR products were subjected to melt curve analysis and sequencing to confirm amplification of the correct target. A standard curve was used to calculate the PCR efficiency of each target. At each time point biological replicates of n = 3 and technical replicates of n≥3 were used. Data was analyzed using qbasePLUS v2.1 (Biogazelle) software. A standard curve was used to estimate the PCR efficiency of each primer set and individual samples were normalized to GAPDH. For within tissue and across breed comparisons samples were normalized to *N expression levels and tested for significance using an unpaired t-test. For all across breed and across tissue comparisons all sample groups were normalized to the group with the lowest expression level within a single gene. For the across breed and across tissue comparisons a one-way ANOVA was used to calculate significance values. Error bars in all gene expression figures represent 95% confidence intervals.

## Supporting Information

Figure S1 Five SNPs show fixed heterozygosity in FM chickens. A diverse breed panel was genotyped using the 60K Chicken iSelect chip. SNPs within the 483 kb region identified in the mapping population are shown. The five SNPs displaying fixed heterozygosity in *FM individuals are indicated with an arrow and define a 75 kb region. Orange is homozygous reference allele, yellow is heterozygous, blue is homozygous mutant allele, and black is missing data.
(PDF)

Figure S2 Agarose gel image of FM PCR diagnostic test products. Results of the PCR diagnostic test for both duplication junction points (assay A and B). All three genotypic classes are shown (*FM/*FM, *FM/*N and *N/*N), although the results of the heterozygote are identical to the homozygous mutant due to the retention of all wild-type sequences within the duplicated and inverted structure of the mutant allele.
(PDF)

Figure S3 Gene expression in FM adult skin and muscle tissue. Across breed and tissue gene expression analysis by SYBR Green qPCR of *FM (Silkie breed) tissue and *N (New Hampshire breed) calibrated to glyceraldehyde 3-phosphate dehydrogenase (*GAPDH*). Error bars indicate 95% confidence intervals and sample groups with significant (p<0.05) differences in expression level within a single gene are indicated by different superscripts. The data table below the graph shows the numerical value of the plotted values.
(PDF)

Table S1 Sequence variation at the 5′ breakpoint of Duplication 1 in FM chickens. The base pair coordinates across the top row are relative to the breakpoint at position 0. The first individual, J16, is FM*N and is used as the reference sequence. A “.” indicates the same allele as the reference and empty cells are missing data. Four different populations of Silkie chickens are shown, with each bird verified to be homozygous for the duplication associated with FM by genomic qPCR.
(PDF)

Table S2 Sequence variation at the 3′ breakpoint of Duplication 1 in FM chickens. The base pair coordinates across the top row are relative to the breakpoint at position 0. The first individual, J16, is FM*N and is used as the reference sequence. A “.” indicates the same allele as the reference and empty cells are missing data. Four different populations of Silkie chickens are shown, with each bird verified to be homozygous for the duplication associated with FM by genomic qPCR.
(PDF)

Table S3 Sequence variation at the 5′ breakpoint of Duplication 2 in FM chickens. The base pair coordinates across the top row are relative to the breakpoint at position 0. The first individual, J16, is FM*N and is used as the reference sequence. A “.” indicates the same allele as the reference and empty cells are missing data. Four different populations of Silkie chickens are shown, with each bird verified to be homozygous for the duplication associated with FM by genomic qPCR.
(PDF)

Table S4 Sequence variation at the 3′ breakpoint of Duplication 2 in FM chickens. The base pair coordinates across the top row are relative to the breakpoint at position 0. The first individual, J16, is FM*N and is used as the reference sequence. A “.” indicates the same allele as the reference and empty cells are missing data. Four different populations of Silkie chickens are shown, with each bird verified to be homozygous for the duplication associated with FM by genomic qPCR.
(PDF)

Table S5 Lack of sequence variation at the 5′ junction of Duplication 1 and Duplication 2 in FM chickens. The base pair coordinates across the top row are relative to the breakpoint at position 0. The first individual, G7, is used as the reference sequence. A “.” indicates the same allele as the reference and empty cells are missing data. Four different populations of Silkie chickens are shown, with each bird verified to be homozygous for the duplication associated with FM by genomic qPCR.
(PDF)

Table S6 Lack of sequence variation at the 3′ junction of Duplication 1 and Duplication 2 in FM chickens. The base pair coordinates across the top row are relative to the breakpoint at position 0. The first individual, G7, is used as the reference sequence. A “.” indicates the same allele as the reference and empty cells are missing data. Four different populations of Silkie chickens are shown, with each bird verified to be homozygous for the duplication associated with FM by genomic qPCR.
(PDF)

Table S7 Primer sequences for genomic DNA PCR assays.
(PDF)

Table S8 Primer sequences for cDNA qPCR assays.
(PDF)
