# Supplementary material for: A Complex Genomic Rearrangement Involving the Endothelin 3 Locus Causes Dermal Hyperpigmentation in the Chicken
Source: PLoS Genet. 2011 Dec 22;7(12):e1002412. doi: 10.1371/journal.pgen.1002412 (PMC3245302; doi:10.1371/journal.pgen.1002412)

Figure S1. Five SNPs show fixed heterozygosity in FM chickens.

A diverse breed panel was genotyped using the 60K Chicken iSelect chip. SNPs within the 483 kb region identified in the mapping population are shown. The five SNPs displaying fixed heterozygosity in *FM individuals are indicated with an arrow and define a 75 kb region. Orange is homozygous reference allele, yellow is heterozygous, blue is homozygous mutant allele, and black is missing data.

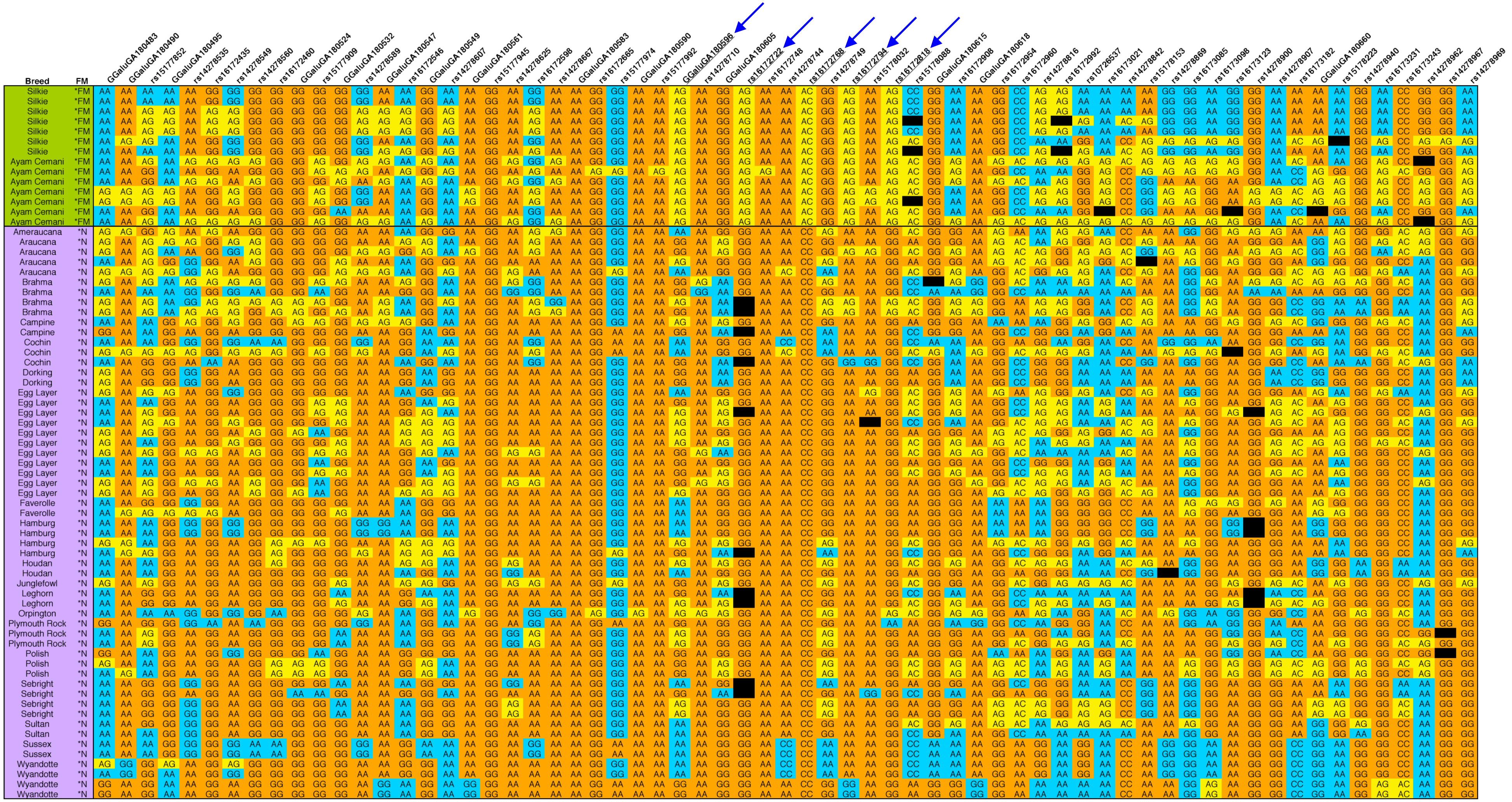

Supplement: Figure S1 — Five SNPs show fixed heterozygosity in FM chickens. A diverse breed panel was genotyped using the 60K Chicken iSelect chip. SNPs within the 483 kb region identified in the mapping population are shown. The five SNPs displaying fixed heterozygosity in *FM individuals are indicated with an arrow and define a 75 kb region. Orange is homozygous reference allele, yellow is heterozygous, blue is homozygous mutant allele, and black is missing data. (PDF) [file pgen.1002412.s001.pdf]
