# Supplementary material for: A Complex Genomic Rearrangement Involving the Endothelin 3 Locus Causes Dermal Hyperpigmentation in the Chicken
Source: PLoS Genet. 2011 Dec 22;7(12):e1002412. doi: 10.1371/journal.pgen.1002412 (PMC3245302; doi:10.1371/journal.pgen.1002412)

Figure S2. Agarose gel image of FM PCR diagnostic test products.
Results of the PCR diagnostic test for both duplication junction points (assay A and B). All three genotypic classes are shown (*FM/ *FM, *FM/ *N and *N/ *N), although the results of the heterozygote are identical to the homozygous mutant due to the retention of all wild-type sequences within the duplicated and inverted structure of the mutant allele.

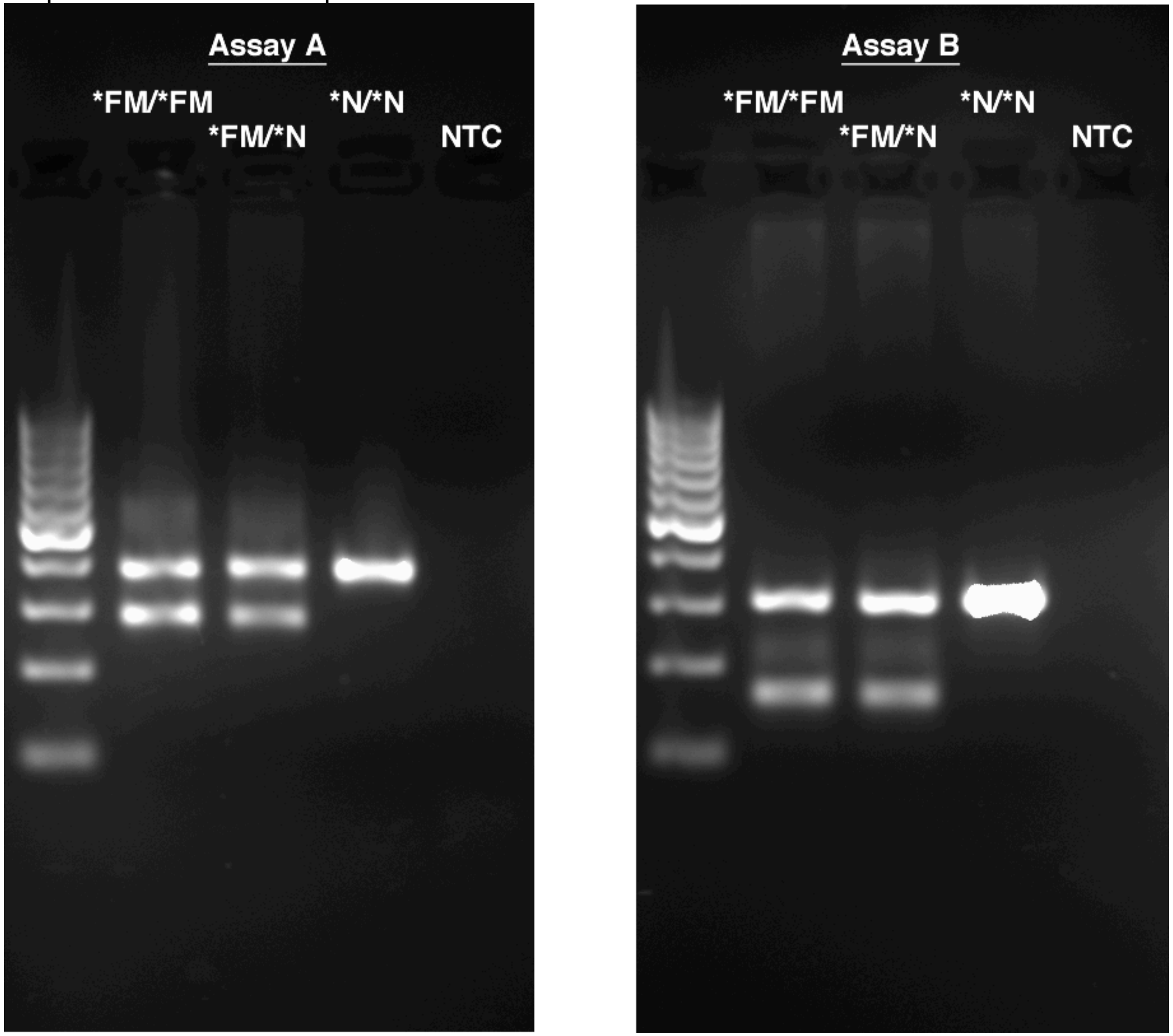

Supplement: Figure S2 — Agarose gel image of FM PCR diagnostic test products. Results of the PCR diagnostic test for both duplication junction points (assay A and B). All three genotypic classes are shown (*FM/*FM, *FM/*N and *N/*N), although the results of the heterozygote are identical to the homozygous mutant due to the retention of all wild-type sequences within the duplicated and inverted structure of the mutant allele. (PDF) [file pgen.1002412.s002.pdf]
