# Supplementary material for: A Complex Genomic Rearrangement Involving the Endothelin 3 Locus Causes Dermal Hyperpigmentation in the Chicken
Source: PLoS Genet. 2011 Dec 22;7(12):e1002412. doi: 10.1371/journal.pgen.1002412 (PMC3245302; doi:10.1371/journal.pgen.1002412)
Supplement: Figure S3 — Gene expression in FM adult skin and muscle tissue. Across breed and tissue gene expression analysis by SYBR Green qPCR of *FM (Silkie breed) tissue and *N (New Hampshire breed) calibrated to glyceraldehyde 3-phosphate dehydrogenase (GAPDH). Error bars indicate 95% confidence intervals and sample groups with significant (p<0.05) differences in expression level within a single gene are indicated by different superscripts. The data table below the graph shows the numerical value of the plotted values. (PDF) [file pgen.1002412.s003.pdf]

Figure S3. Gene expression in FM adult skin and muscle tissue.
Across breed and tissue gene expression analysis by SYBR Green qPCR of **FM* (Silkie breed) tissue and **N* (New Hampshire breed) calibrated to glyceraldehyde 3-phosphate dehydrogenase (*GAPDH*). Error bars indicate 95% confidence intervals and sample groups with significant ($P<0.05$) differences in expression level are indicated by different superscripts. The data table below the graph shows the numerical value of the plotted values.

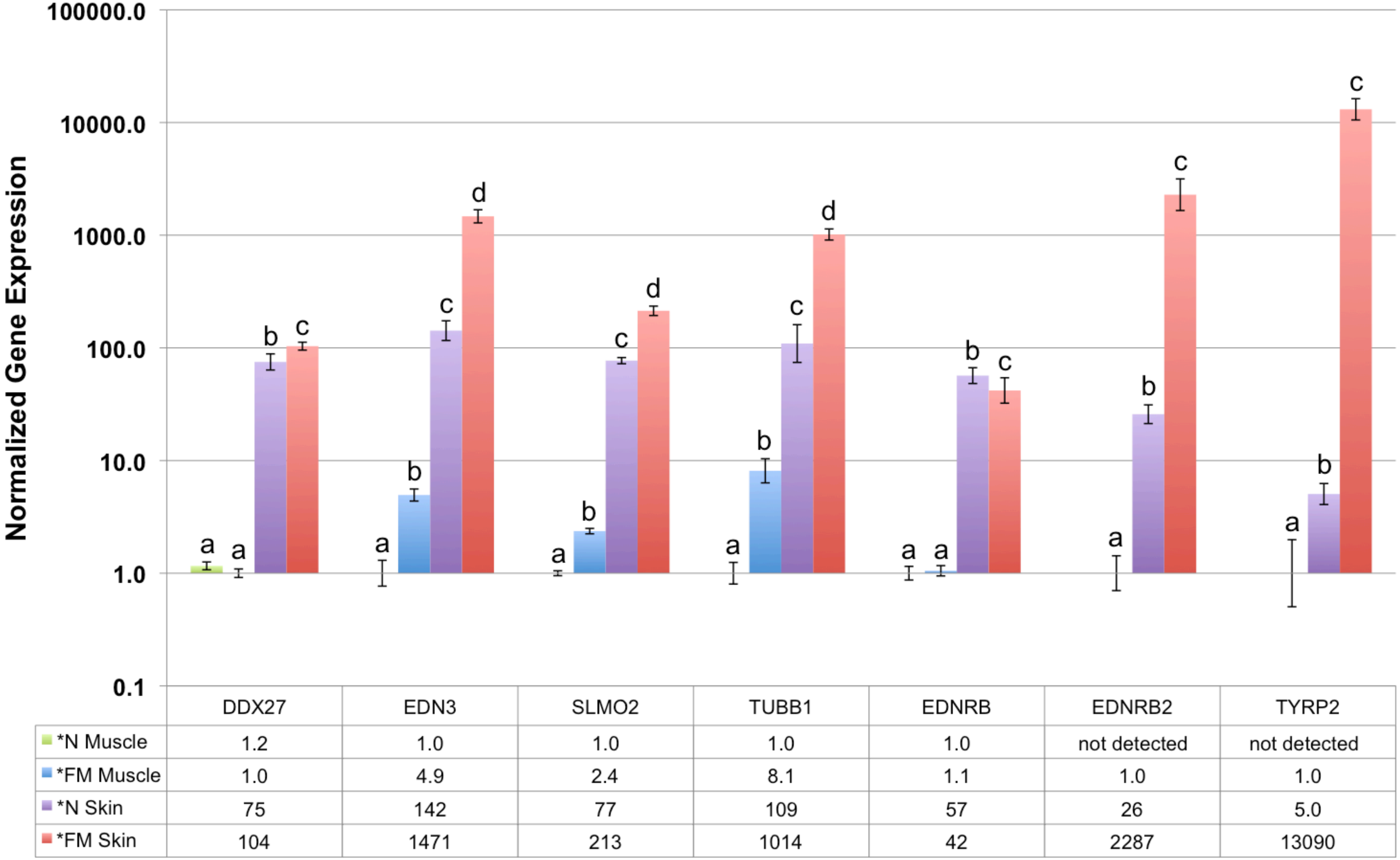

	DDX27	EDN3	SLMO2	TUBB1	EDNRB	EDNRB2	TYRP2
*N Muscle	1.2	1.0	1.0	1.0	1.0	not detected	not detected
*FM Muscle	1.0	4.9	2.4	8.1	1.1	1.0	1.0
*N Skin	75	142	77	109	57	26	5.0
*FM Skin	104	1471	213	1014	42	2287	13090
