# Supplementary material for: A Complex Genomic Rearrangement Involving the Endothelin 3 Locus Causes Dermal Hyperpigmentation in the Chicken
Source: PLoS Genet. 2011 Dec 22;7(12):e1002412. doi: 10.1371/journal.pgen.1002412 (PMC3245302; doi:10.1371/journal.pgen.1002412)
Supplement: Table S3 — Sequence variation at the 5′ breakpoint of Duplication 2 in FM chickens. The base pair coordinates across the top row are relative to the breakpoint at position 0. The first individual, J16, is FM*N and is used as the reference sequence. A “.” indicates the same allele as the reference and empty cells are missing data. Four different populations of Silkie chickens are shown, with each bird verified to be homozygous for the duplication associated with FM by genomic qPCR. (PDF) [file pgen.1002412.s006.pdf]

Table S3. Sequence variation at the 5' breakpoint of Duplication 2 in FM chickens.
The base pair coordinates across the top row are relative to the breakpoint at position 0. The first individual, J16, is FM*N and is used as the reference sequence. A "." indicates the same allele as the reference and empty cells are missing data. Four different populations of Silkie chickens are shown, with each bird verified to be homozygous for the duplication associated with FM by genomic qPCR.

Sample ID	Breed	-455	-213	-104	-25	373
J16	Polish	A	G	T	T	C
G7	Silkie-A	.	.	.	C	.
G8	Silkie-A	.	.	.	.	.
G9	Silkie-A	.	.	.	het	.
G10	Silkie-A	.	.	.	het	.
1746	Silkie-B	.	.	.	het	.
1747	Silkie-B	.	.	.	het	.
1748	Silkie-B	.	.	.	het	.
417	Silkie-C	.	.	.	C	.
434	Silkie-C	.	.	.	het	.
438	Silkie-C	.	.	.	C	.
1	Silkie-D	.	.	.	het	.
5	Silkie-D	.	.	.	.	.
7	Silkie-D	.	.	.	het	.
