# Supplementary material for: A Complex Genomic Rearrangement Involving the Endothelin 3 Locus Causes Dermal Hyperpigmentation in the Chicken
Source: PLoS Genet. 2011 Dec 22;7(12):e1002412. doi: 10.1371/journal.pgen.1002412 (PMC3245302; doi:10.1371/journal.pgen.1002412)
Supplement: Table S5 — Lack of sequence variation at the 5′ junction of Duplication 1 and Duplication 2 in FM chickens. The base pair coordinates across the top row are relative to the breakpoint at position 0. The first individual, G7, is used as the reference sequence. A “.” indicates the same allele as the reference and empty cells are missing data. Four different populations of Silkie chickens are shown, with each bird verified to be homozygous for the duplication associated with FM by genomic qPCR. (PDF) [file pgen.1002412.s008.pdf]

Table S5. Lack of sequence variation at the 5' junction of Duplication 1 and Duplication 2 in FM chickens.
The base pair coordinates across the top row are relative to the breakpoint at position 0. The first individual, G7, is used as the reference sequence. A "." indicates the same allele as the reference and empty cells are missing data. Four different populations of Silkie chickens are shown, with each bird verified to be homozygous for the duplication associated with FM by genomic qPCR.

Sample ID	Breed	-360	420
G7	Silkie-A	A	A
G8	Silkie-A	.	.
G9	Silkie-A	.	.
G10	Silkie-A	.	.
1746	Silkie-B	.	.
1747	Silkie-B	.	.
1748	Silkie-B	.	.
417	Silkie-C	.	.
434	Silkie-C	.	.
438	Silkie-C	.	.
1	Silkie-D	.	.
5	Silkie-D	.	.
7	Silkie-D	.	.
