# Supplementary material for: A Complex Genomic Rearrangement Involving the Endothelin 3 Locus Causes Dermal Hyperpigmentation in the Chicken
Source: PLoS Genet. 2011 Dec 22;7(12):e1002412. doi: 10.1371/journal.pgen.1002412 (PMC3245302; doi:10.1371/journal.pgen.1002412)
Supplement: Table S7 — Primer sequences for genomic DNA PCR assays. (PDF) [file pgen.1002412.s010.pdf]

Table S7. Primer sequences for genomic DNA PCR assays.

ID	Name	Purpose	Orientation	Chr.	Position (bp)	Primer Sequence
4		Genomic qPCR	F	1	68,335,770	CCGTCATCCAGAGCACTTAC
5		Genomic qPCR	TaqMan Probe	1	68,335,794	TCAAAGGCGAGGAGCCCC
6		Genomic qPCR	R	1	68,335,886	GGTCATCCTCTTCCTCATCATA
236		Breakpoint Sequencing	F	20	10,716,685	AGACCGGCTTCAAGAATGTCTC
232		Diagnostic Test Assay A	F	20	10,717,082	AGAAACAAGGGTCAAGGTGAGC
		BREAKPOINT		20	10,717,294	
234		Diagnostic Test Assay A	R	20	10,717,460	TGGATCATTGGAGGAAGTGTTG
196	Dup1_5'	Breakpoint Sequencing	R	20	10,717,500	GGAAGCCTACAATCTCCAGCAT
237		Breakpoint Sequencing	R	20	10,717,721	ACACTCCACATGTCCCTCTGAA
1		Genomic qPCR	F	20	10,748,149	GGCAGGAATTGAACCTCATT
2		Genomic qPCR	TaqMan Probe	20	10,748,207	CAGCACGGGTCGCTCAGC
3		Genomic qPCR	R	20	10,748,254	TTGTTTGCACTCCAAAGCTC
238		Breakpoint Sequencing	F	20	10,845,625	CAATGCAGGAGCAGATAACCAC
152	Dup1_3'	Breakpoint Sequencing	F	20	10,845,625	CAATGCAGGAGCAGATAACCAC
197		Diagnostic Test Assay B	F	20	10,846,172	GCAGCCTTTATTATTGCGTGTG
		BREAKPOINT		20	10,846,232	
239		Breakpoint Sequencing	R	20	10,846,681	CAACCAACCCAGTAACCACAAG
240		Breakpoint Sequencing	F	20	11,262,328	TGTGTGGCTCAGCTTTGTATCA
		BREAKPOINT		20	11,262,904	
200	Dup2_5'	Diagnostic Test Assay A	R	20	11,263,016	GGGATGGCTCTCACATAAAAGG
241		Breakpoint Sequencing	R	20	11,263,388	CCCAATGTAGCAGCACACTTCT
35		Genomic qPCR	F	20	11,398,002	ACACCGGGGTTTCTGTATGT
36		Genomic qPCR	TaqMan Probe	20	11,398,077	TCCGAGGTTATTCCCAGATGGG
37		Genomic qPCR	R	20	11,398,118	CAGACAGGCCTCGACAGATA
242		Breakpoint Sequencing	F	20	11,434,817	CAGGTCCTGTTTTCCAAACCTC
201		Diagnostic Test Assay B	F	20	11,435,158	CTTGGCTCAGATATTCGCCTCT
153	Dup2_5'	Breakpoint Sequencing	F	20	11,435,158	CTTGGCTCAGATATTCGCCTCT
		BREAKPOINT		20	11,435,256	
202		Diagnostic Test Assay B	R	20	11,435,459	AGGCACAGTCTGGCACATTAAA
243		Breakpoint Sequencing	R	20	11,435,822	GAACAAACCCATGCCATTTTCT
