# Supplementary material for: A Complex Genomic Rearrangement Involving the Endothelin 3 Locus Causes Dermal Hyperpigmentation in the Chicken
Source: PLoS Genet. 2011 Dec 22;7(12):e1002412. doi: 10.1371/journal.pgen.1002412 (PMC3245302; doi:10.1371/journal.pgen.1002412)
Supplement: Table S8 — Primer sequences for cDNA qPCR assays. (PDF) [file pgen.1002412.s011.pdf]

Table S8. Primer sequences for cDNA qPCR assays.

ID	Gene	GenBank ID	Orientation	Chr.	Position (bp)	Product Size (bp)	Primer Sequence
304	GAPDH	NM_204305	F	1		108	GGTGAAAGTCGGAGTCAACGG
305	GAPDH	NM_204305	R	1		108	TCGATGAAGGGATCATTGATGGC
308	EDNRB	NM_001001127	F	1	158,008,082	140	CACCCCACACAGAAAACATC
309	EDNRB	NM_001001127	R	1	158,008,545	140	TTCTTTCGTAACATCTCACAGG
310	EDNRB2	NM_204120	F	4	11,262,828	189	ACCGTCTTCTGCCTCGTG
311	EDNRB2	NM_204120	R	4	11,263,325	189	GAGAGCCACAGGATTGATGC
316	TYRP2	NM_204935	F	1	150,689,148	154	CCTCAAGTTCTTCAACCAGAG
317	TYRP2	NM_204935	R	1	150,697,447	154	CCTCTCCTCCACTGTCAAG
318	DDX27	NM_001006293	F	20	10,704,938	117	TTGAGGATCGGAAACGCTTG
319	DDX27	NM_001006293	R	20	10,704,564	117	GATAGCCCACATTCCCCACT
320	TUBB1	NM_205445	F	20	10,844,351	127	CTTCAGATTGGCCAGTGTGG
321	TUBB1	NM_205445	R	20	10,842,599	127	CGTTAATTCGCTCAAGCTGC
322	SLMO2	NM_001030866	F	20	10,826,184	164	GGCTCGTCTATAAACCACACC
323	SLMO2	NM_001030866	R	20	10,827,722	164	TACCCATTCCAATGCTTCACG
324	EDN3	XM_001231487	F	20	10,765,765	97	CATCTGGATCAACACCCCAG
325	EDN3	XM_001231487	R	20	10,753,923	97	ACTTTGATTCTGGCCCGTAG
